# Human Standing Posture Motion Evaluation by the Visual Simulation of Multi-Directional Sea-Waves

**DOI:** 10.3390/s22155884

**Published:** 2022-08-06

**Authors:** Renon Doine, Takanori Sakamaki

**Affiliations:** 1 Department of Clinical Engineering, Faculty of Human Care at Makuhari, Tohto University, Chiba 261-0021, Japan; 2 Department of Informatics and Mechanical Engineering, National Institute of Technology, Toba College, Toba 517-8501, Japan

**Keywords:** fatigue, visual simulator, human standing posture, center of pressure, anticipatory postural adjustments

## Abstract

Crew fatigue from standing posture motion, caused by ship motion, can lead to marine accidents. Therefore, the mechanism of fatigue in crew members ought to be elucidated. The standing posture of humans is maintained by postural state detection through the visual, vestibular, and somatosensory systems. Humans can adjust their posture through corrective postural reactions (CPR) generated after anticipatory postural adjustments (APAs) by using information from these sensory systems. APAs refer to skills acquired by learning from past motions and perturbations and are prepared by the central nervous system based on visual information before the actual perturbation occurs. We hypothesized that APAs would decrease fatigue in crew members by stabilizing their standing posture motions. We aimed to clarify the human standing posture control influenced by APAs based on visual information. To this end, we presented wave images with different wave directions to the participants using a visual simulator and analyzed their standing posture motion. We found that the participants stabilized their standing posture based on the projected wave directions. This showed that the participants predicted ship motion from the wave images and controlled their center of pressure (COP) through APAs. Individual differences in standing postural motion may indicate the subjective variation of APAs based on individual experiences. This study was limited to males aged 20–23 years. To generalize this study, randomized controlled trials should be performed with participants of multiple age groups, including men and women.

## 1. Introduction

In 2019, the total annual trade volume of imports and exports of Japan exceeded 900 million tons, of which 99.6% constituted maritime trade. In 2020, 1954 marine accidents occurred, demonstrating the criticality of preventing them. Human error accounted for 74% of these accidents [1]. The fatigue of crew members is the primary cause of accidents. Therefore, the mechanism of crew member fatigue ought to be elucidated.

Crew members constantly control their posture to maintain their standing posture against ship motion, which causes physical fatigue [2]. On measuring the physical fatigue of crew members in terms of energy expenditure, pitch-and-roll motion, generated in a ship motion simulator, was identified as increasing fatigue [3]. The pitch-and-heeling motion of the ship was also found to affect the crew’s energy expenditure [4]. Our previous studies focused on fatigue based on the postural control of crew members and analyzed their energy expenditure and standing posture motion [5,6,7,8,9]. The international standard ISO6954 evaluates the habitability of ships based on ship motion; however, it ignores physiological indicators [10].

The standing posture of humans is stabilized by corrective postural reactions (CPR), generated after anticipatory postural adjustments (APAs), using information from the visual, vestibular, and somatosensory systems [11]. Specifically, the visual system detects postures based on visual information; the vestibular system senses the orientation of the head with respect to gravity, depending on the balance between the vestibule and the semicircular canal of the inner ear; the somatosensory system detects postural movements based on muscle and joint movements [11,12]. APAs refer to skills acquired by learning from past motions and perturbations and are prepared by the central nervous system based on visual information before the actual perturbation occurs [11]. CPR generates an adjustment after an actual perturbation [11]. Standing posture motion caused by APAs and CPR is observed as a change in the center of pressure (COP) [11].

The control system for the human standing posture depends strongly on visual information [11]. Thus, analyzing the effects of standing posture motion triggered by visual information on the crew members is crucial. In previous studies, we analyzed the energy expenditure of the participants and their standing posture motion triggered by the wave images presented using a ship-handling simulator. No significant differences were detected between the energy expenditure of participants exposed to images with and without waves [13,14,15]. The standing posture motion may have been caused by the APAs of the participants based on the visual information.

We hypothesized that APAs would decrease fatigue in crew members by stabilizing their standing posture. The current study aimed to clarify the human standing posture control resulting from APAs based on visual information. Thus, we presented wave images with different wave directions to the participants using a visual simulator and analyzed their standing posture motions.

## 2. Methods

### 2.1. Participants

For the present study, the inclusion criteria were an age between 20 and 23 years and male sex. Table 1 presents the details of the seven participants involved in the experiment. The procedures were explained to the participants before initiating the experiment, and they provided written informed consent. To prevent the effects of eating, exercising, and sleeping on energy expenditure, the participants were instructed to get sufficient sleep the previous night. They were also instructed to abstain from eating, drinking anything except water, and engaging in intense exercise within 4 h before the start of the experiment.

### 2.2. Design

**Figure 1:**
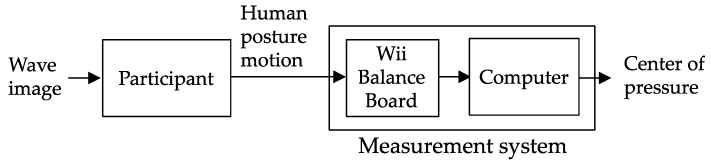
Schematic of the study.

This case study analyzed the relationship between the wave direction projected by a visual simulator and the direction of human standing posture motion. The intervention for the participants was based on visual information. Figure 1 shows a schematic of the study. Wave images were presented to seven participants using a visual simulator, and the COP was monitored to determine the human posture motion. Each participant balanced themselves on a Wii Balance Board (RVL-021; Nintendo, Kyoto, Japan).

Four image patterns were presented to seven participants using a visual simulator: an image without waves (Pattern 1), an image with 180° waves moving from front to rear (Pattern 2), an image with 135° waves moving from front left to rear right (Pattern 3), and an image with 90° waves moving from left to right (Pattern 4). One set of experiments consisted of a 15 min break in the sitting posture with the image of Pattern 1 and a 15 min intervention in the standing posture with one of the four image patterns. In the first, second, third, and fourth sets of experiments, the images of Patterns 1, 3, 2, and 4 were presented to the participants during the intervention phase, respectively. We instructed the participants to stand naturally by placing their feet shoulder-width apart during the intervention phase.

### 2.3. Data Collection and Processing

The standing posture motion caused by the APAs was observed as a change in the COP. In this study, COP was measured using a Wii Balance Board. The specifications of the Wii Balance Board are listed in Table 2. The Wii Balance Board has four strain-gauge-based load sensors capable of obtaining movement data in the COP and communicating wirelessly with a computer via Bluetooth. A systematic review indicated that the Wii Balance Board can provide data that are concurrently valid with typical commercial force platforms. In addition, the board has reliability characteristics similar to those of force platforms for static standing [16]. The intraclass correlation coefficient (ICC) is a statistical test of reliability. Four studies reported the excellent reliability of the Wii Balance Board (ICC = 0.76 to 0.94) [17,18,19,20].

The COP was measured at a sampling rate of 100 Hz. A low-pass filter with a cut-off frequency of 1 Hz was applied to the COP signals to eliminate noise. The characteristics of the COP signals were evaluated in terms of the total length of the COP, AP/ML, which is the ratio of the AP length to the ML length of the COP, and the relationship between the ML and AP lengths of the COP, using scatter diagrams and regression lines. The sample size for the total length of the COG and AP/ML was set to seventeen, respectively, to design an effect size of at least 1.0, and the level of statistical power was at least 0.8. The effect size indicates Cohen’s d. The statistical power was calculated using the significance level, sample size, and effect size.

### 2.4. Experimental Environment

#### 2.4.1. Visual Simulator

**Figure 2:**
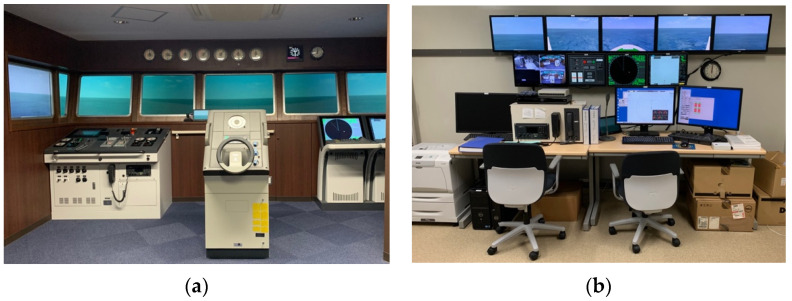
Ship handling simulator: (**a**) wheelhouse; (**b**) setting room of the simulating condition.

A ship-handling simulator was used as a visual simulator. A ship-handling simulator reproduces a vessel-maneuvering environment as close as possible to an actual wheelhouse by combining navigation equipment and computer graphics technology. Figure 2 shows the ship-handling simulator used in this study at the National Institute of Technology, Toba College, Mie, Japan. The simulator was based on the International Convention on Standards of Training, Certification, and Watchkeeping for Seafarers (STCW). The horizontal and vertical viewing angles, centered on a gyrocompass installed at the center of the wheelhouse, located 3 m from the screen, were 225° and 30°, respectively.

#### 2.4.2. Specifications of the Simulated Ship and Wave Images

**Figure 3:**
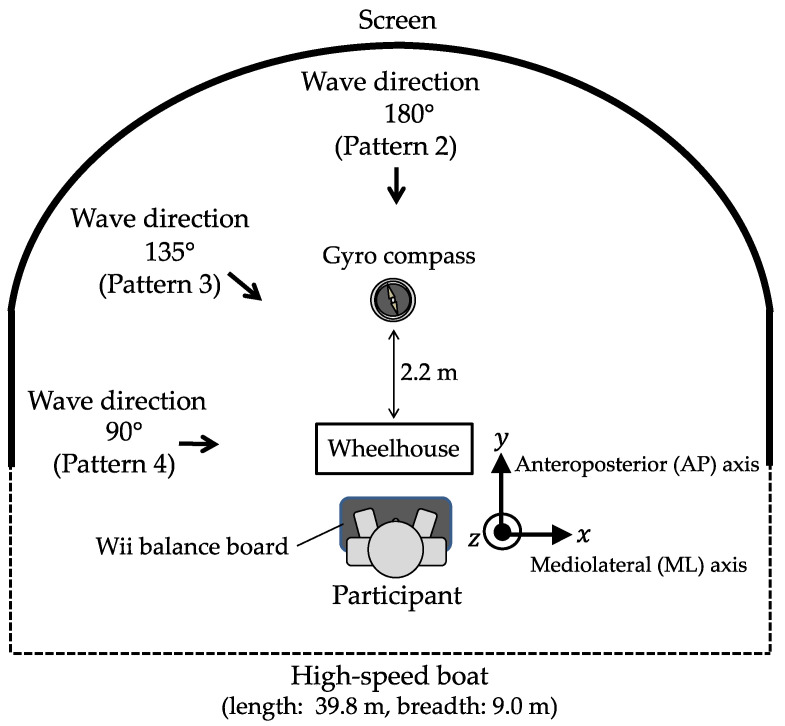
Wave directions with respect to the participant’s position.

**Figure 4:**
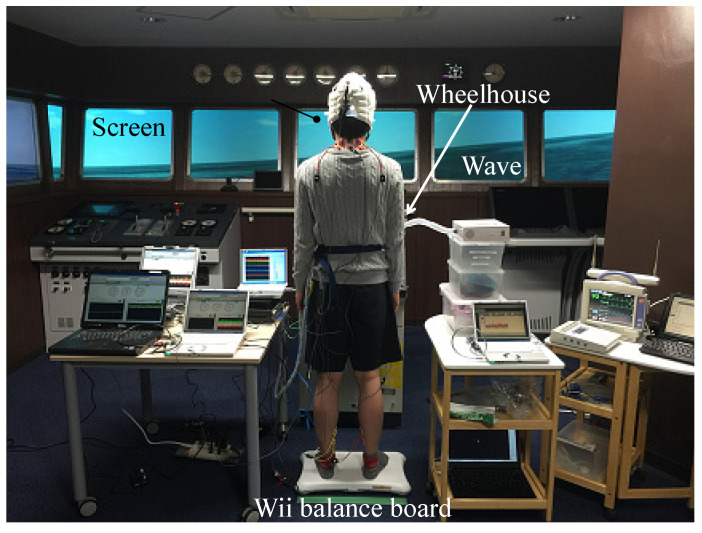
Experimental setup.

**Figure 5:**
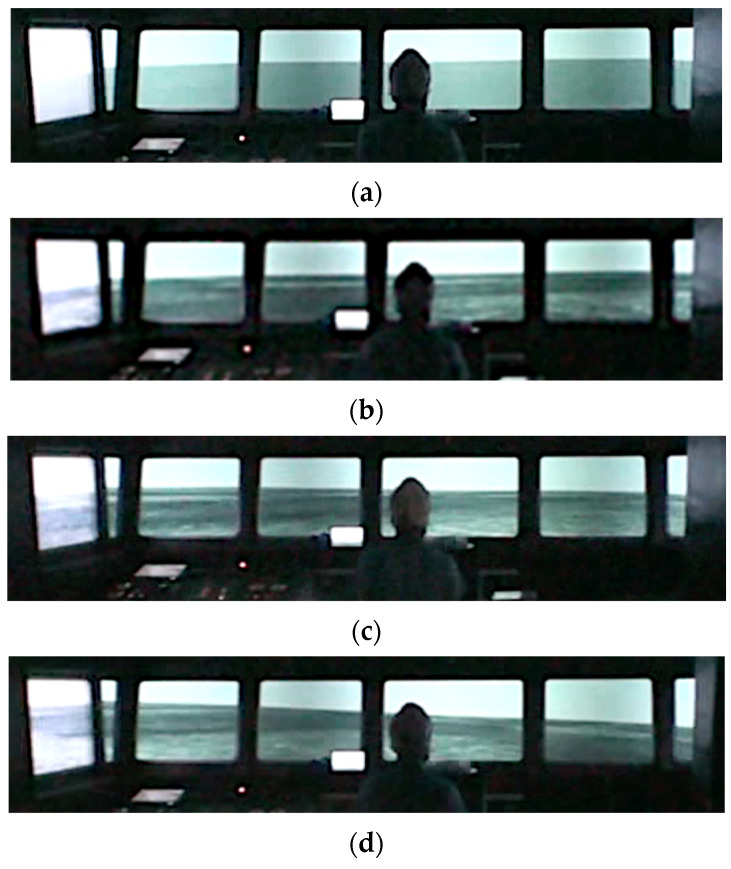
Simulated wave images: (**a**) Pattern 1 (images without waves); (**b**) Pattern 2 (wave direction: 180°); (**c**) Pattern 3 (wave direction: 135°); (**d**) Pattern 4 (wave direction: 90°).

A high-speed boat (length: 39.8 m, breadth: 9.00 m) was used for the entire pattern. The course and speed were 0° and 15 kn, respectively. The sea wind speed was set to zero. Table 3 lists the four wave image patterns in different directions. Pattern 1 was defined as an image without waves. For Patterns 2, 3, and 4, the wave directions were set to 180°, 135°, and 90°, respectively. The wave height and wave period were set to 3 m and 8 s, respectively; the condition was confirmed to occur in the posture motion in previous research using the visual simulator [13]. Figure 3 illustrates the wave directions with respect to the participant’s position. The participants were positioned behind the steering wheel at a distance of 2.2 m from the gyrocompass located at the center of the screen and instructed to look at the screen (bowside). Figure 4 depicts the experimental setup. The cables connecting the participant to the measurement instruments were fixed to a belt on the waist of the participant to avoid limiting their standing posture motion. Figure 5 shows the simulated wave images used for each experimental condition.

Table 4 lists the motion of the simulated high-speed boat. The pitch motion (rotational motion about the *x*-axis) was the largest in Pattern 2. The roll motion (rotational motion about the *y*-axis) was the largest in Pattern 4.

### 2.5. Evaluation Indicators

**Figure 6:**
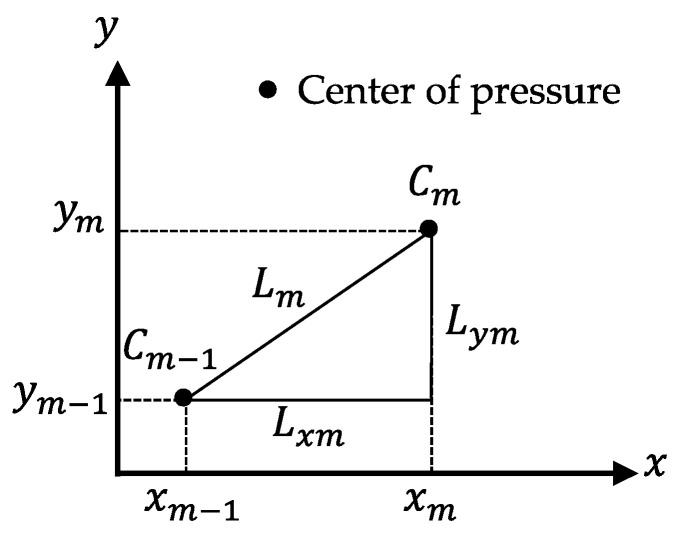
Conceptual diagram of the center of pressure (COP) length.

Figure 6 presents a conceptual diagram of the COP length.

The coordinate value $C_{m}$ ($x_{m}$,$y_{m}$) indicates the mth COP sample, and $C_{m-1}$($x_{m-1}$,$y_{m-1}$) represents the (m − 1)th COP sample. $L_{m}$ denotes the distance from $C_{m-1}$ to $C_{m}$. The total length of the COP is denoted by $L_{M}$ and is calculated as follows:

(1) $$L_{M}=\sum_{m=1}^{M}L_{m}=\overset{M}{\underset{m=1}{\sum }}\sqrt{{\left(x_{m}-x_{m-1}\right)}^{2}+{\left(y_{m}-y_{m-1}\right)}^{2}}.$$

$L_{xm}$ denotes the distance from $x_{m-1}$ to $x_{m}$. The ML lengths of the COP are denoted by $L_{xM}$, which is calculated as

(2) $$L_{xM}=\sum_{m=1}^{M}L_{xm}=\sum_{m=1}^{M}\left|x_{m}-x_{m-1}\right|\;.$$

$L_{ym}$ denotes the distance from $y_{m-1}$ to $y_{m}$. The AP length of the COP is denoted by $L_{yM}$, which is calculated as

(3) $$\;L_{yM}=\sum_{m=1}^{M}L_{ym}=\sum_{m=1}^{M}\left|y_{m}-y_{m-1}\right|\;.$$

AP/ML is the ratio of the AP length to the ML length of the COP. The ratio was calculated as follows:

(4) $$AP/ML=\frac{L_{yM}}{L_{xM}}.$$

M was set to 3200. Data for more than 30 s were necessary to investigate the standing posture [21]. This period included four periodic wave images.

Seventeen data points were calculated for each wave pattern for $L_{M}$ and AP/ML, which were common to all participants. $L_{M}$ or AP/ML, corresponding to the participants changing the position of their feet on the Wii Balance Board or shifting the position of their COP to one foot, was removed from the dataset of the 17 data points for each wave pattern. Statistical outliers were removed from the dataset of the 17 data points for each wave pattern.

The characteristics of the standing posture motion of each participant were analyzed by calculating the mean values and standard deviations of the 17 $L_{M}$ or AP/ML data points for each wave pattern. Additionally, the characteristics of the standing posture motion of all participants were analyzed by calculating the mean values and standard deviations of $L_{M}$ or AP/ML collected from all participants for each wave pattern and by using scatter diagrams and regression lines of $L_{xM}$ and $L_{yM}$. Tukey’s method [22], which is a parametric multiple comparison method, was employed to examine significant differences in the total length of the COP ($L_{M}$) and ratio (AP/ML) between each wave image pattern. The significance level was set at *p* < 0.05.

## 3. Results

**Figure 7:**
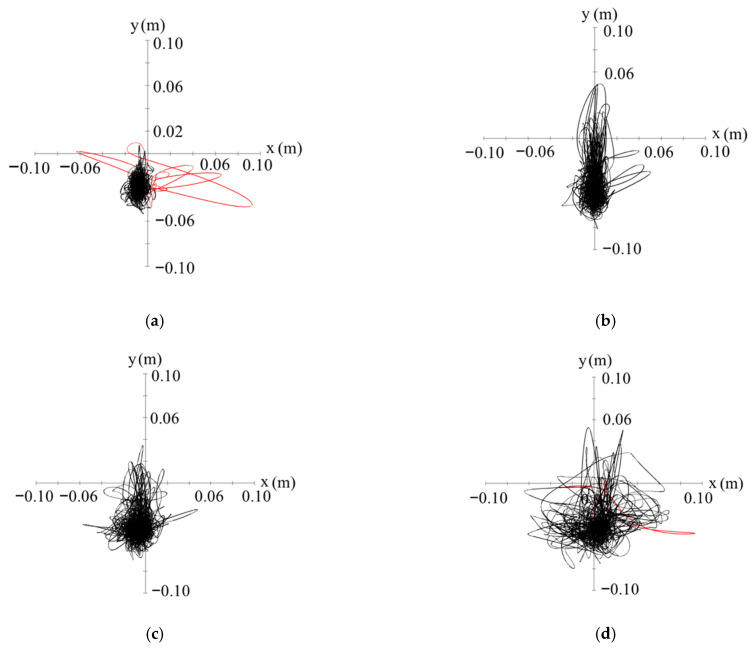
Example of the center of pressure (COP) locus (participant D): (**a**) Pattern 1; (**b**) Pattern 2 (wave direction: 180°); (**c**) Pattern 3 (wave direction: 135°); (**d**) Pattern 4 (wave direction: 90°).

Figure 7 shows an example of the COP locus. The sampled data represented by the red lines indicate that the participant changed the position of their feet on the Wii Balance Board (Figure 7). $L_{M}$ and AP/ML included in the sampled data, shown by red lines, were removed from the dataset of the 17 $L_{M}$ or AP/ML data points for each wave pattern. The *AP* and *ML* motions of the COP differed from those presented in Figure 7b–d depending on the difference in the wave direction.

**Figure 8:**
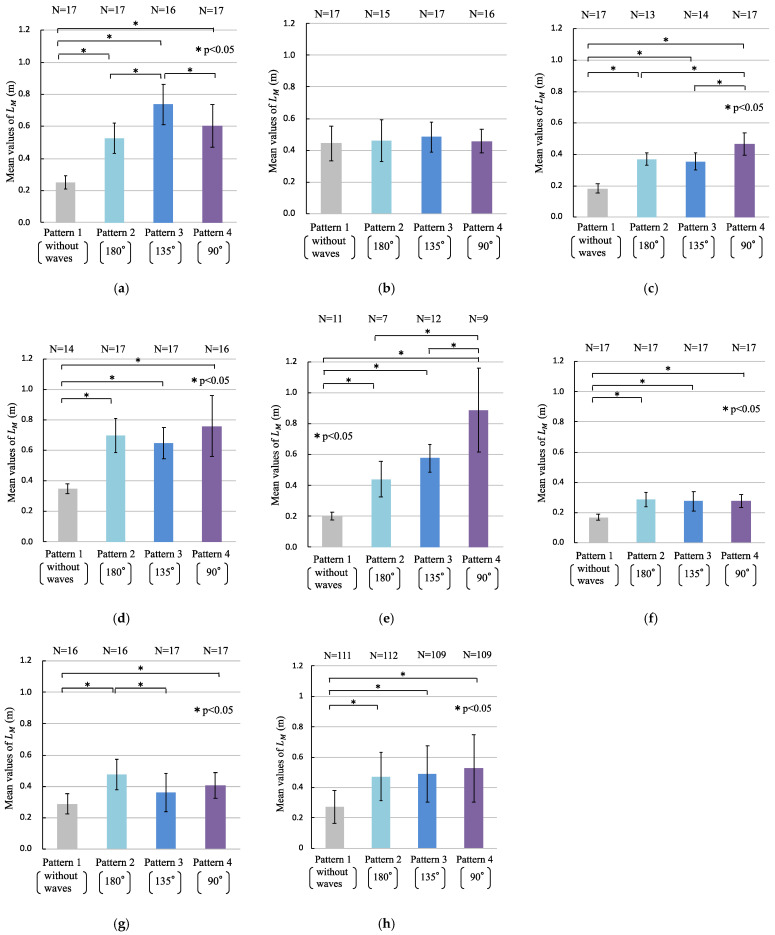
Total length of the center of pressure (COP) ($L_{M}$): (**a**) participant A; (**b**) participant B; (**c**) participant C; (**d**) participant D; (**e**) participant E; (**f**) participant F; (**g**) participant G; (**h**) all participants. N is the number of valid $L_{M}$. The asterisk (*) indicates a significant difference (* *p* < 0.05).

Figure 8a–g illustrate the mean values and standard deviations of $L_{M}$ for each participant. Figure 8h shows the mean values and standard deviations of $L_{M}$ for all participants. N represents the number of valid $L_{M}$ that were removed from the data of the participants changing or shifting their foot positions on the Wii Balance Board or the actual data of statistical outliers from the dataset of the 17 data points for each wave pattern. Moreover, significant differences were detected in the total COP length between Pattern 1 and the other patterns (*p* < 0.05) (Figure 8h). Significant differences were detected in the total length of the COP length of participants, as shown in Figure 8a,c,d–f, as well as between the same pairs of wave patterns, as shown in Figure 8h (*p* < 0.05). As shown in Figure 8b, no significant differences were detected in the total COP between all the pairs of wave patterns. The levels of the effect size of the multiple comparison method, which show significant differences, were 1.03 to 4.60. The levels of the statistical power of the multiple comparison method, which show significant differences, were 0.82 to 1.00.

**Figure 9:**
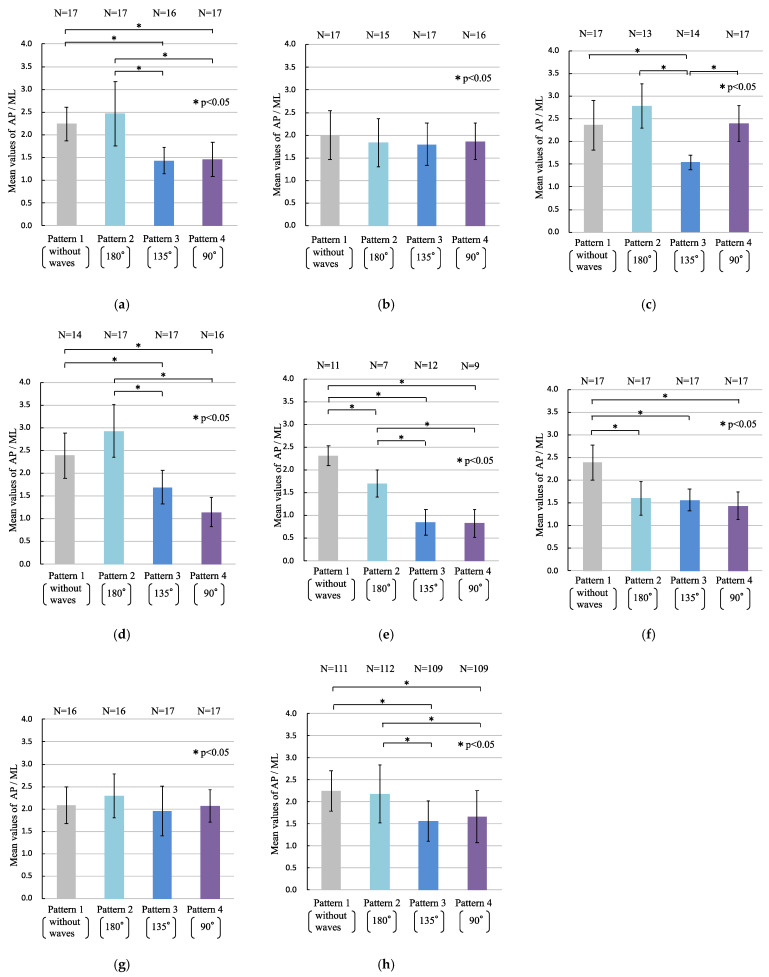
Ratio of the anteroposterior (AP) length to the mediolateral (ML) length of the center of pressure (COP) (AP/ML): (**a**) participant A; (**b**) participant B; (**c**) participant C; (**d**) participant D; (**e**) participant E; (**f**) participant F; (**g**) participant G; (**h**) all participants. N is the number of valid R. The asterisk (*) indicates a significant difference (* *p* < 0.05).

Figure 9a–g present the mean values and standard deviations of AP/ML for each participant. Figure 9h shows the mean values and standard deviations of AP/ML for all participants. N denotes the number of valid AP/ML that were removed from the data of the participants changing or shifting their feet position on the Wii Balance Board or the actual data of statistical outliers from the dataset of 17 data points for each wave pattern. Significant differences were detected in the ratios between Patterns 1 and 3, Patterns 1 and 4, Patterns 2 and 3, and Patterns 2 and 4 (*p* < 0.05) (Figure 9h). Significant differences were detected in the ratio of the AP to ML length of the COP of participants, as shown in Figure 9a,d,e, as well as between the same pairs of patterns, as shown in Figure 9h (*p* < 0.05). As shown in Figure 9b,g, no significant differences were detected in the ratio between all pairs of wave patterns. The levels of the effect size of the multiple comparison method, which show significant differences, were 1.08 to 5.82. The level of the statistical power of the multiple comparison method, which shows significant differences, was 1.00.

**Figure 10:**
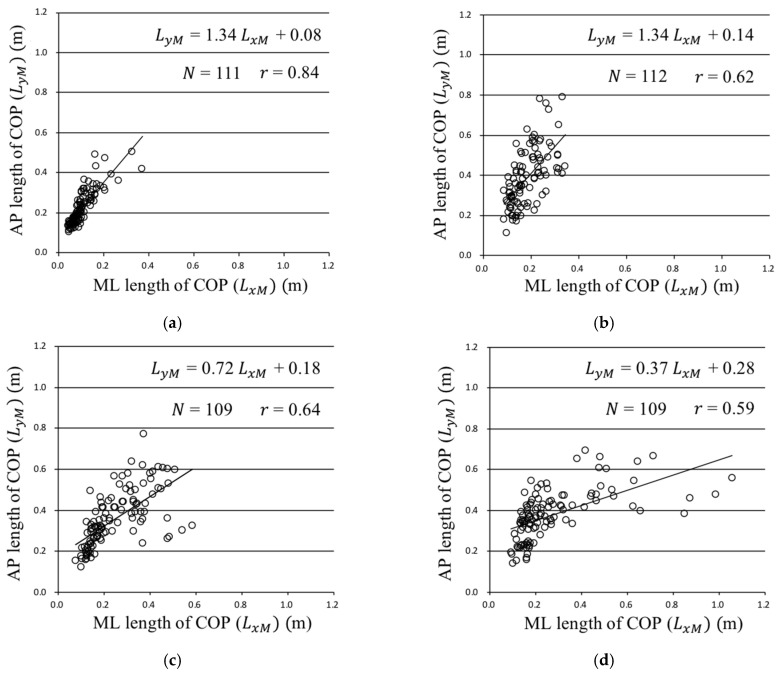
Scatter diagram and regression lines of the mediolateral (ML) and anteroposterior (AP) lengths of the ratio shown in Figure 9h: (**a**) Pattern 1; (**b**) Pattern 2 (wave direction: 180°); (**c**) Pattern 3 (wave direction: 135°); (**d**) Pattern 4 (wave direction: 90°).

Figure 10 shows the scatter diagrams and regression lines of the *ML* ($L_{xM}$) and *AP* ($L_{yM}$) lengths of the ratio presented in Figure 9h for each wave pattern. The slope of the regression line under Patterns 1 and 2 was larger than 1.0; therefore, the *AP* COP motion was dominant compared with the *ML* COP motion. In contrast, the slope of the regression line under Patterns 3 and 4 was smaller than 1.0; therefore, the *ML* COP motion was dominant compared with the *AP* COP motion. Figure 10b–d show that the slope of the regression line decreased as the wave direction changed from 180° to 90°.

## 4. Discussion

The effects of unidirectional visual information on human standing posture motion have been previously studied [23,24,25]. These studies focused on the relationship between a unidirectional moving screen or moving object and the human standing posture motion while participants watched a moving object [13,14,15,23,24,25].

In the current research, only sea-waves were shown to the participants using a visual simulator, and the experimental results reflected the effect of visual information on human standing posture. Figure 8h shows that the total length of the COP motion in the participants watching the wave motion (Patterns 2–4) was larger than that in those not watching the wave motion (Pattern 1). Figure 9h shows that a significant difference in the COP motion was observed depending on whether the sea-waves were incident on the images on the screen from the lateral direction, primarily because the COP motion under Patterns 1 and 2 could be distinguished from that under Patterns 3 and 4. Figure 10 illustrates that the *ML* motion of the COP increased with the wave directions changing to 180°, 135°, and 90°. Most participants exhibited standing posture motion. The sense of the participants’ posture motions was not an illusion of body movement but real body posture motions based on visual sensation. For sea-waves approaching head-on, the participants maintained their posture by moving back and forth, whereas they stood firmly on the deck for waves approaching laterally. In the quiet standing posture, the COG in the *AP* direction is controlled by the ankle dorsiflexors and plantar flexors, whereas the COG in the *ML* direction is controlled by the hip abductors and adductors [26,27,28,29]. Thus, humans maintain their posture based on visual information through *AP* and *ML* motions, owing to the structure of their lower limbs. In the current study, we found that the standing postures of the participants stabilized themselves according to the wave directions projected by a visual simulator. This showed that the participants predicted ship motion from the wave images and controlled their COP through the APAs.

In the experimental results (Figure 8 and Figure 9), individual differences were observed in the standing postural motions based on visual information. These differences may indicate the APA ability of each participant. Figure 8b shows negligible differences in the participants’ standing postural motion irrespective of the waves. Figure 9b,g also show no significant difference in the participants’ standing postural motion between the directions of the waves. APAs are categorized as natural APAs and learned APAs [30]. When a participant experiences a situation in which they can control their standing posture using only CPR against a ship’s motion, APAs may not occur for similar ship motions. The analysis of human standing postural motion based on the differences between the wave directions projected by a visual simulator using COP can be used to evaluate the physical aptitude of crews. Further experiments are required to confirm this hypothesis.

The assumed role of APAs is to minimize perturbations in standing posture, and APAs are prepared by the central nervous system before CPR occurs. Therefore, visual information is potentially effective for the predictive control of the standing posture. Most crew members, except navigators, cannot see ocean waves or the tilt of a ship, as they work in confined spaces. Consequently, they control their posture against ship motion by using vestibular and proprioceptive information, without relying on vision, which may increase fatigue. By setting a screen to project the sea-waves inboard, the crew members can develop visual APAs for standing posture adjustments, which may reduce fatigue. The evaluation approach of human standing posture motion using the multi-directional visual information presented in this study is a new method for evaluating APAs. Patients with Parkinson’s disease have been shown to exhibit abnormalities in programming APAs, which contribute to their postural instability [31,32]. Thus, evaluating the APAs of patients with postural accommodation disorder using the evaluation method of this study may assist physical therapists in performing effective rehabilitation.

The limitation of this study is that the results cannot be applied to female populations and to age groups other than 20–23 years. To generalize this method, randomized controlled trials ought to be performed with participants of multiple age groups, including men and women.

## 5. Conclusions

The direction of the standing postural motion of the participants depended on the direction of sea-waves, which was projected by a visual simulator and was not an illusion of body movement. Individual differences in standing postural motion may indicate the ability of APAs for each participant based on their experience.

We would like to develop a method for evaluating the adaptability of crew members by conducting additional experiments to test this hypothesis. This method can be applied to the realization of a system for evaluating adaptability to crew training. The fatigue of crew members inboard may be reduced by APAs through the development of a wave motion presentation system.

## Figures and Tables

**Table 1:** Participants involved in the experiment.

Participant	Age	Height (cm)	Weight (kg)	Sex
A *	23	165	66	male
B *	22	177	76	male
C	21	180	65	male
D *	22	185	92	male
E	20	178	78	male
F	20	161	48	male
G *	20	180	77	male

Asterisk (*) indicates that the participant has a marine license.

**Table 2:** Specifications of the Wii Balance Board.

Description		Specification
Manufacturer		Nintendo
Product family		Wii
Type name		RVL-021
Communications standard		Bluetooth ver.1.2
Wireless frequency		2.4 GHz
Sampling interval		0.01 s
Weight limit		136 kg
Measured precision	0–67 kg	±800 g
	68–99 kg	±1.2 kg
	100–136 kg	±2.0 kg
Product weight		3.6 kg
Outside dimension	Width	511 mm
	Height	316 mm
	Depth	53.2 mm

**Table 3:** Wave image patterns.

	Pattern 1	Pattern 2	Pattern 3	Pattern 4
Direction	0°	180°	135°	90°
Height	0 m	3 m	3 m	3 m
Period	0 s	8 s	8 s	8 s

**Table 4:** Motion of the simulated high-speed boat.

	Pattern 1	Pattern 2	Pattern 3	Pattern 4
Roll	0.00°	−1.56°–1.55°	−9.47°–9.09°	−13.20°–12.69°
Pitch	0.00°	−10.78°–10.26°	−8.77°–7.50°	−5.93°–5.87°
Yaw	0.00°	−0.02°–0.02°	−0.11°–0.11°	−0.13°–0.13°

## Data Availability

The data supporting the findings of this study are available from the corresponding author (S.T.) upon reasonable request.

## References

[1] The Japan Ship Owners’ Mutual Protection & Indemnity (2021). 4M4 (5) E Analysis: Analysis of Accident Cases.

[2] American Bureau of Shipping and Lamar University (2015). Vessel Motion Effects on Crew.

[3] Wertheim A.H., Heus R., Vrijkotte T.G.M. (1994). Energy expenditure, physical work load and postural control during walking on a moving platform. TNO Def. Res..

[4] Breidahl T., Christensen M., Jepsen J.R., Johansen J.P., Omland Ø. (2013). The influence of ship movements on the energy expenditure of fishermen: A study during a North Sea voyage in calm weather. Int. Marit. Health.

[5] Renon D., Takanori S., Hiroaki S., Masamitsu I., Akihiko H., Yasuhiro F. (2015). Analysis of the standing postural motion of passengers against ship motion. Life Support.

[6] Renon D., Takanori S., Hiroaki S., Akihiko H. (2017). The behavior of passengers’ postural control against ship motion on a small marine craft using surface electromyogram. J. Jpn. Inst. Navig..

[7] Renon D., Takanori S., Hiroaki S., Masamitsu I., Akihiko H., Yasuhiro F. (2015). The exercise load of passengers’ postural control against ship motion using human energy expenditure. J. Adv. Biomed. Eng..

[8] Takanori S., Renon D., Hiroaki S., Nobuo O. (2016). Exercise load and physical motion on standing posture in a small marine craft and motion system. J. Jpn. Inst. Navig..

[9] Renon D., Fujio M., Hiroaki S., Takanori S. (2019). Effects of standing postural motions on human energy expenditures in a small marine craft. J. Jpn. Inst. Navig..

[10] (2000). Mechanical Vibration-Guidelines for the Measurement, Reporting and Evaluation of Vibration with Regard to Habitability on Passenger and Merchant Ships.

[11] Mark L. (2008). Latash. Neurophysiological Basis of Movement.

[12] Maranesi E., Fioretti S., Ghetti G.G., Rabini R.A., Burattini L., Mercante O., Di Nardo F. (2016). The surface electromyographic evaluation of the Functional Reach in elderly subjects. J. Electromyogr. Kinesiol..

[13] Renon D., Takanori S., Hiroaki S., Akihiko H. (2017). Effects of the projected images of a ship handling simulator on trainees’ standing postural motion. J. Jpn. Inst. Navig..

[14] Renon D., Hiroaki S., Akihiko H., Takanori S. (2019). Effects of wave direction difference projected by a ship handling simulator on human standing postural motion. J. Jpn. Inst. Navig..

[15] Takanori S., Renon D. Effects of wave images on standing posture of ship crew. Proceedings of the 14th International Symposium on Advances in Technology Education (ISATE).

[16] Ross A.C., Benjamin F.M., Yong-Hao P., Kelly J.B. (2018). Reliability and validity of the Wii Balance Board for assessment of standing balance: A systematic review. Gait Posture.

[17] Bower K.J., McGinley J.L., Miller K.J., Clark R.A. (2014). Instrumented static and dynamic balance assessment after stroke using Wii Balance Boards: Reliability and association with clinical tests. PLoS ONE.

[18] Larsen L.R., Jørgensen M.G., Junge T., Juul-Kristensen B., Wedderkopp N. (2014). Field assessment of balance in 10 to 14 year old children, reproducibility and validity of the Nintendo Wii board. BMC Pediatr..

[19] Monteiro R.S., Ferreira A.S., Puell V.N., Lattari E., Machado S., Vaghetti C.A.O., Silva E.B. (2015). Wii balance board: Reliability and clinical use in assessment of balance in healthy elderly women. CNS Neurol. Disord. Drug Targets.

[20] Park D.S., Lee G. (2014). Validity and reliability of balance assessment software using the Nintendo Wii Balance Board: Usability and validation. J. NeuroEng. Rehabil..

[21] Kaoru I., Hitoshi M., Miho F. (1997). Collection of data for healthy subjects in stabilometry. Equilib. Res..

[22] Aczel S. (2009). Complete Business Statistics.

[23] Cedrick T.B., Elise F., Michael A.R., Benoît G.B., Thomas A.S. (2006). Motion sickness preceded by unstable displacements of the center of pressure. Hum. Mov. Sci..

[24] Sébastien J.V., Moira B.F., Gina M.A., Thomas A.S. (2008). Postural instability and motion sickness in a virtual moving room. Hum. Factors.

[25] Joshua L.H., Srikant V., Nicholas S. (2014). Gaze and posture coordinate differently with the complexity of visual stimulus motion. Exp. Brain Res..

[26] David A.W., Prince F., Frank J.S., Powell C., Zabjek K.F. (1996). Unified theory regarding A/P and M/L balance in quiet stance. J. Neurophysiol..

[27] David A.W. (2009). Biomechanics and Motor Control of Human Movement.

[28] Fay H., Nashner L.M. (1986). Central programming of postural movements: Adaptation to altered support surface configurations. Neurophysiology.

[29] William H.G., David A.W., James S.F., Allan L.A. (2004). Kinematic and kinetic validity of the inverted pendulum model in quiet standing. Gait Posture.

[30] Oleg K., Irina S., Vera T., Marat I. (2008). Anticipatory postural adjustment: The role of motor cortex in the natural and learned bimanual unloading. Exp. Brain Res..

[31] Andrea C.D.L., Daniel B.C., Mariana P.N., Catarina C.B., Alana X.B., Raymundo M.D.A.N., Carla S.B., Egberto R.B., Rajal G.C., Fay B.H. (2020). Brain networks associated with anticipatory postural adjustments in Parkinson’s disease patients with freezing of gait. NeuroImage Clin..

[32] Jacobs J.V., Lou J.S., Kraakevik J.A., Horak F.B. (2009). The supplementary motor area contributes to the timing of the anticipatory postural adjustment during step initiation in participants with and without Parkinson’s disease. Neuroscience.

